# Profiling gut microbiota and bile acid metabolism in critically ill children

**DOI:** 10.1038/s41598-022-13640-0

**Published:** 2022-06-21

**Authors:** Iain Robert Louis Kean, Joseph Wagner, Anisha Wijeyesekera, Marcus De Goffau, Sarah Thurston, John A. Clark, Deborah K. White, Jenna Ridout, Shruti Agrawal, Riaz Kayani, Roddy O’Donnell, Padmanabhan Ramnarayan, Mark J. Peters, Nigel Klein, Elaine Holmes, Julian Parkhill, Stephen Baker, Nazima Pathan

**Affiliations:** 1 Department of Paediatrics, University of Cambridge, Cambridge, United Kingdom; 2 The Peter Doherty Institute for Infection and Immunity, Melbourne Health, Melbourne, Australia; 3 Wellcome Sanger Institute, Cambridge, United Kingdom; 4 Department of Food and Nutritional Sciences, University of Reading, Reading, United Kingdom; 5 Department of Experimental Vascular Medicine, Amsterdam UMC Location University of Amsterdam, Amsterdam, The Netherlands; 6 Department of Veterinary Medicine, University of Cambridge, Cambridge, United Kingdom; 7 Department of Haematology, University of Cambridge, Cambridge, United Kingdom; 8 EACH, Milton, Cambridge, United Kingdom; 9 Cambridge University Hospitals NHS Foundation Trust, Addenbrooke’s Hospital, Cambridge, United Kingdom; 10 Paediatric Intensive Care Unit, Great Ormond Street Hospital for Children NHS Foundation Trust, London, United Kingdom; 11 St Mary’s Hospital, London, United Kingdom; 12 UCL Great Ormond Street Institute of Child Health, London, United Kingdom; 13 Section of Biomolecular Medicine, Division of Systems Medicine, Department of Metabolism, Digestion and Reproduction, Imperial College London, London, United Kingdom; 14 Cambridge Institute of Therapeutic Immunology and Infectious Disease, Jeffrey Cheah Biomedical Centre, University of Cambridge, Cambridge, United Kingdom; 15 Department of Medicine, University of Cambridge, Cambridge, United Kingdom

**Keywords:** Clinical microbiology, Microbial communities, Microbiota, Paediatric research

## Abstract

Broad-spectrum antimicrobial use during the treatment of critical illness influences gastrointestinal fermentation endpoints, host immune response and metabolic activity including the conversion of primary to secondary bile acids. We previously observed reduced fermentation capacity in the faecal microbiota of critically ill children upon hospital admission. Here, we further explore the timecourse of the relationship between the microbiome and bile acid profile in faecal samples collected from critically ill children. The microbiome was assayed by sequencing of the 16S rRNA gene, and faecal water bile acids were measured by liquid chromatography mass spectrometry. In comparison to admission faecal samples, members of the *Lachnospiraceae* recovered during the late-acute phase (days 8–10) of hospitalisation. Patients with infections had a lower proportion of *Lachnospiraceae* in their gut microbiota than controls and patients with primary admitting diagnoses. Keystone species linked to ecological recovery were observed to decline with the length of PICU admission. These species were further suppressed in patients with systemic infection, respiratory failure, and undergoing surgery. Bile acid composition recovers quickly after intervention for critical illness which may be aided by the compositional shift in *Lachnospiraceae*. Our findings suggest gut microbiota recovery can be readily assessed via measurement of faecal bile acids.

## Introduction

The establishment of a healthy gut microbiota in early life is considered to be protective against a range of allergies and communicable and non-communicable diseases^1–8^. Multiple factors, such as delivery method, feeding, diet, illness, age, and antimicrobial use in early life, can impact on the establishment and maintenance a healthy gut microbiota^1,2,8,9^. The administration of broad-spectrum antimicrobials is often necessary during hospitalisation for critical illness, which reduces the diversity of the gut microbiota^3^. The effects of broad-spectrum antimicrobial therapy on the microbiota, and the subsequent recovery process, have been well documented^10–12^. However, critical illness can have profound effects on feeding and energy intake^13^, and respiratory complications may lead to low oxygen saturation and subsequent hypoxia, inducing disruption of the gut microbiota^14^. Furthermore, changes in the structure of the microbiota may exacerbate the disease state or alter the course of recovery^3,12,15^. The body of evidence identifying illness as both a cause and effect of an altered microbiota is becoming more extensive^9^.

Bile acids shape the composition of the microbiota and correspondingly the structure of the microbiota contributes to the metabolism of the bile acids^16^. Notably, the conversion from primary to secondary bile acids requires a cascade of enzymatic modifications incorporating a 7α-dehydratase enzyme expressed by a range of Gram-positive bacteria^17,18^. Further metabolism of bile acids by intestinal bacteria enhances passive reabsorption by the intestine; therefore, the microbiota also contribute to the recycling of bile acids^19^. Secondary bile acids can also inhibit intestinal pathogens and have been shown to restrict the germination and sporulation of *Clostridioides*^20,21^. The relationship between the microbiome and disease is complex; altered bile acid profiles are associated with diseases such as metabolic syndrome and colon cancer, which may also be exacerbated by the microbiota^22–26^. The western diet has been associated with higher levels of taurocholic acid, hydrogen sulphide and deoxycholic acid, which have been linked to ulcerative colitis and colon cancer^24,27–30^. Bile acids modulate the farnesoid X receptor and G-protein-coupled bile acid receptor, with both receptors playing active roles in glucose and lipid homeostasis as well as insulin sensitivity^25,26^.

We previously characterised the functional capacity of the intestinal microbiome by exploiting multi-compartmental metabolic profiling^10^, and observed a shift in the short chain fatty acid and faecal bile acids profiles in critically ill children compared to healthy age-matched controls. Our previous work examined the acute changes in faecal microbiome in children admitted to the Paediatric Intensive Care Unit (PICU) for critical illness. Here we examine differences in the microbiome of critically ill children on ventilators during different stages of hospitalization for critical illness and the associated impact on total faecal bile acid composition.

## Results

### Demographics

We examined samples from 70 mechanically ventilated critically ill children and 51 age-matched healthy controls. The critically ill children were aged between 1 and 16.9 years with a median age of 4.1 years (inter-quartile range (IQR); 8.5 years). The healthy controls were aged between 1 and 15.9 years, with a median age of 5.8 years (IQR; 2.6 years) (*p* = 0.31). The median weight of the critically ill children was 15.7 kg, (IQR; 13.9 kg); the healthy controls had a median weight of 20.4 kg (IQR; 9 kg) (*p* = 0.005). The median Weight for Age Z-score for patients was -0.135 (IQR; 2.313). The median Weight for Age Z-score for the healthy controls was 0.420 (IQR; 0.820). Our patient cohort was 55.7% female (39/70), and our control cohort was 55.0% female (28/51). The median number of PICU free days within 30 days of admission in the critically ill children was 22 (IQR; 8 days) and the median number of days free of invasive mechanical ventilation within 30 days of admission was 26 days (IQR; 4.5 days). The survival rate was 96%. Further demographics are shown in Table S1 and Table S2.

### Bile acids

**Figure 1:**
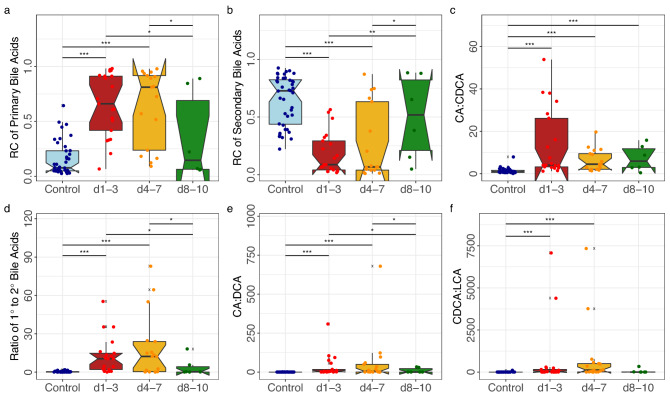
Primary and secondary bile acids (**a**) primary bile acids (Cholic acid (CA), Chenodeoxycholic acid (CDCA), Glycochenodeoxycholic acid (GCDCA), and Taurocholic acid (TCA)) in faecal water measured by LC–MS as a ratio of total measured bile acid. (**b**) secondary bile acids (Deoxycholic Acid (DCA), Lithocholic acid (LCA), Isolithocholic acid (ILCA), Ursodeoxycholic acid (UDCA), 3α-Hydroxy,12-Ketolithocholic acid (3a-H,12-KLCA), Taurohyocholic acid (THCA), and Glycoursodeoxycholic acid (GUDCA)) in faecal water measured by LC–MS as a ratio of total measured bile acid. A significant reduction to the relative concentration of total secondary bile acids versus the total measured bile acid pool was observed for patients sampled at day 1–3 and day 4–7 compared to the control. The median proportion of these secondary bile acids at days 8–10 was comparable to that of the aged-matched controls, but we observed a significant increase of patient bile acids at days 8–10 above the median for patients at day 1–3. (**c**) the ratio of CA to CDCA, two species of primary bile acids in the human gut. The control samples had a median CA:CDCA ratio of 0.9; the median CA:CDCA ratios on days 1–3, 4–7, an 8–10 in the faecal samples of the critically patients were 4.4, 4.4, and 5.9, respectively. (**d**) Ratio of primary bile acids to secondary bile acids. (**e**) ratio of CA to DCA in faecal water. The ratio of CA to DCA was elevated in across all patient timepoints compared to the control. Patient samples collected on days 8–10 had lower ratios of CA:DCA than patients at day 1–3. (**f**) ratio of CDCA to LCA and ILCA. CDCA:LCA ratios were increased compared to controls in patient samples from day 1–3 and day 4–7, returning to control levels at days 8–10. Conover-Iman significance ***p ≤ 0.001, **p ≤ 0.01, *p ≤ 0.05, *p* > 0.05 = not shown.

We analysed the composition of faecal bile acids and the relative concentrations of different bile acid groups using LC–MS on 43 stool samples collected from 39 critically ill children and 40 samples from healthy controls. The critically ill children sampled on day 1–3 and day 4–7 had significantly higher concentrations of primary bile acids in their faeces compared than the control group (*p* < 0.001, *p* < 0.001) (Fig. 1a). Additionally, the concentration of primary bile acids was significantly lower at day 8–10 than earlier in the course of admission (day 1–3 samples) (*p* = 0.008). The inverse trend was observed for the secondary bile acids (Fig. 1b).

We examined the ratio of different bile acids. We observed a significant change in the ratio of the two primary bile acids, (cholic acid and chenodeoxycholic acid; CA:CDCA) in all patient sampling timepoints relative to the control (Fig. 1c). The primary/secondary bile acid ratios in critically ill patients were significantly higher at day 1–3 and day 4–7 compared to controls (*p* < 0.001, *p* < 0.001). Conversely, there was a significant reduction in the primary/secondary bile acid ratios in the samples taken on days 8–10 compared to the control (Fig. 1d). The ratios of cholic acid to deoxycholic acid (CA:DCA) measured in patient sample faecal water significantly increased on day 1–3, day 4–7 and days 8–10, compared to the control samples but the median ratio on days 8–10 was significantly lower than the median of day 1–3 and day 4–7 (Fig. 1e). A significant change was also observed in in the ratio of chenodeoxycholic acid to total lithocholic acid (CDCA:LCA) between the control group and day 1–3 and day 4–7 (Fig. 1f).

To assess bile acid differences at different sampling periods, we compared the relative concentration of individual bile acids (Figure S1). Secondary bile acids were frequently more abundant in control samples than at day 1–3 and day 4–7. However, the median relative concentration of each bile acid from patients sampled at day 8–10 shifted towards the median of the control group for the majority of bile acid interrogations (Fig. 1), Non-DCA/LCA bile acids were more abundant in healthy controls; Hydroxy- and Oxo- modifications at carbon 3 and 12 are reversible moieties catalysed by the gut microbiota, in addition to the well-established 7α-dehydratase conversion of CA to DCA and CDCA to LCA/ILCA.

#### Bacterial populations

Children admitted to a PICU are frequently administered broad spectrum antimicrobials soon after admission. In this study, 42/43 (97.7%) PICU patients whose faecal microbiome was assessed by 16S rRNA gene sequencing, received antimicrobial therapy during the course of PICU admission. Applying a Kruskal–Wallis chi-squared test, no significant difference was observed in the number of antimicrobials given to patients across all sampling groups^10^. No compositional difference was observed between male and females in this study (*p* = 0.80).

**Figure 2:**
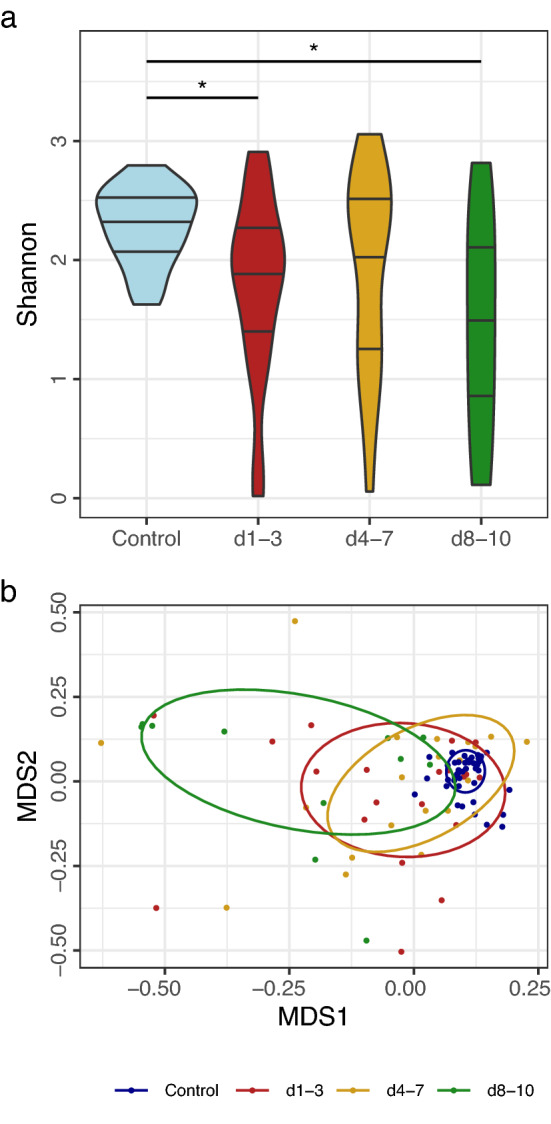
Bacterial diversity, and abundance of beneficial bacteria. (**a**) Shannon diversity of sampling groups, bars indicate 1st 2nd and 3rd quartiles. Due to the broad range of alpha-diversity at day 1–3, day 4–7 and days 8–10, only the median alpha diversity of samples collected at days 8–10 was significantly lower than that of the control group after correcting for multiple comparisons. (**b**) NMDS clustering of beta diversity of bacterial populations compared between sampling groups. PERMANOVA *p*-value = 0.001. Ellipses indicate 60% similarity. Conover-Iman significance ***p ≤ 0.001, **p ≤ 0.01, *p ≤ 0.05, *p* > 0.05 = not shown.

**Figure 3:**
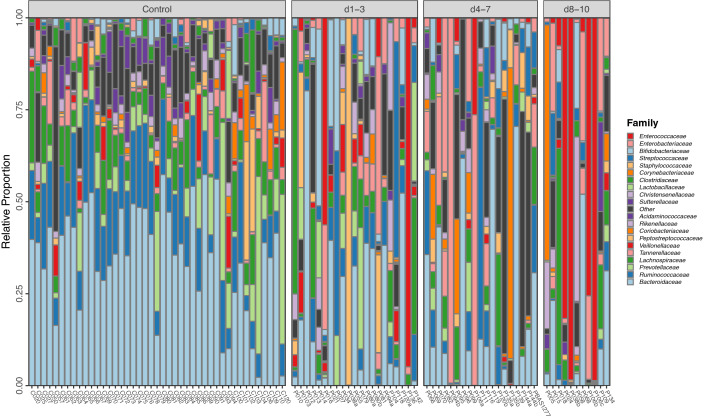
Relative proportion of bacteria identified by 16S rRNA gene sequencing. Bacterial 16S rRNA gene counts are represented as proportional abundance at Family level for each sample. The majority of analysed 16S rRNA gene counts are within 12k to 16k reads. The ten most abundant families in the control microbiomes were the Bacteroidaceae, Ruminococcaceae, Prevotellaceae, Lachnospiraceae, Tannerellaceae, Veillonellacea, Peptostreptococcaceae, Coriobacteriaceae, Rikenellaceae and Acidaminococcaceae. These ten families represent a median of 82.8% (IQR 12.7%) of the control faecal microbiota and a median of 45.3% (IQR 57%) of the patient faecal microbiota. The ten most abundant bacterial families present in the patient faecal microbiome, which were not highly represented in the control samples were the Enterococcaceae, Enterobacteriacea, Bifidobacteriaceae, Streptococcaceae, Staphylococcaceae, Corynebacteriaceae, Clostridiaceae, Lactobacillaceae, Christensenellaceae and the Sutterellaceae. These families represented a median of 7.9% (IQR 8.8%) of the control microbiome, and a median of 31.1% (IQR 64.8%) of the patient samples.

To investigate microbial variation between individuals and groups, alpha and beta diversities were examined. Quantifying alpha diversity using Shannon’s Index, we observed greater median diversity with a narrow range in the control group, compared to all patient sampling time points (Fig. 2a). To determine if groups were compositionally different, beta diversity was calculated using Bray–Curtis dissimilarity. Using NMDS plotting we observed that all patient sample groups significantly diverged from the control group based on Bray–Curtis dissimilarity (Fig. 2b). The difference between groups was significant by PERMANOVA, performed using Adonis from the vegan package in R (*p* = 0.0001). To identify intergroup significance, we applied Tukey’s Honest Significant Differences (HSD) post hoc test to a principal coordinate analysis of the Bray–Curtis dissimilarity matrix, resulting in adjusted *p*-values of < 0.0001 for all pairwise comparisons between the control and patient groups (data not shown). Inter-group analysis using Tukey’s HSD identified no difference between patient samples (*p* > 0.95 for all groups). We observed no difference in microbial populations between male and female patients. The bacterial population profiles of study participants, represented at the Family level, are shown in Fig. 3.

**Figure 4:**
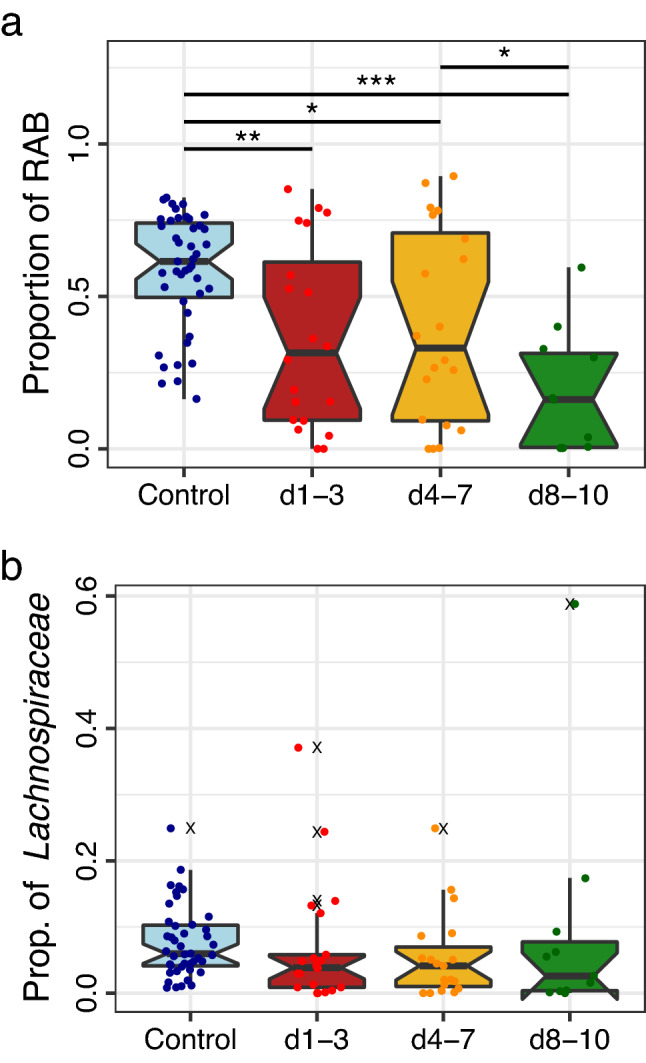
Relative abundance of recovery associated bacteria (RAB) and *Lachnospiraceae*. (**a**) Proportion of RAB present in microbiomes. Patient samples from day 1–3, day 4–7 and days 8–10 were significantly reduced from the median level of the control group. RAB at days 8–10 were also significantly reduced from the median RAB level at day 4–7. (**b**) Proportion of *Lachnospiraceae* present in microbiomes. No significant difference was observed between groups after controlling for multiple comparisons. Conover-Iman significance ***p ≤ 0.001, **p ≤ 0.01, *p ≤ 0.05, *p* > 0.05 = not shown.

Bacteria associated with gut microbiome recovery have previously been characterised in studies observing the fluctuation in microbiota induced by antimicrobial use^12^. Bacterial genera *Alistipes, Bacteroides, Bifidobacterium, Coprococcus, Desulfovibrio, Faecalibacterium, Parabacteroides, Roseburia, Ruminococcus* and *Subdoligranulum* have been previously described as recovery associated bacteria (RAB). Observing these genera as a group, the general proportion of all RAB significantly decrease from control samples across all patient sampling timepoints (Fig. 4a). The loss of RAB was related to the time in which the sample was collected (Figure S2a). No correlation was observed between RAB proportion and number of antimicrobials administered prior to sample collection (ρ[51] =  − 0.058, *p* = 0.6854). Weak negative correlations were observed between RAB proportion and hospital days before sample collection, but this trend was not significant (ρ[51] =  − 0.217, *p* = 0.123). RAB were decreased relative to controls for patients admitted with non-respiratory infections, patients with non-infectious respiratory failure, and surgical patients (Fig. S2b).

We observed a decrease in the proportions of RAB as PICU stay lengthened, with most samples collected at days 8–10 containing < 50% RAB by relative proportion (Fig. 4a). RAB proportion reduced with sample produced, where first samples had higher abundance or RAB than the second or third stool sample collected. (Fig. S2a). RAB were also higher for all admitting diagnosis groups except for Neurological admissions (Fig. S2b). The Individual RAB comparisons are shown in Fig. S3. *Bacteroides* spp. contributed the largest proportion of gut bacteria in the control group (Fig. S3b). The patient group displayed a wide range of *Bacteroides* abundance across sampling ranges; however, the median abundance was significantly below that of the control group for all time frames (Fig. S3b). The majority of patient samples were comprised of < 25% *Bacteroides*. Of the RAB, *Faecalibacterium* spp. and *Ruminococcus* spp. both contributed similarly to the proportion of the control group (Fig. S3). A significant reduction was observed between the control faecal microbiomes and patient faecal microbiomes for both genera (*p* = 0.001, *p* = 0.004, *p* < 0.001). The amount of *Faecalibacterium* during all timepoints in PICU stays was dramatic, with an average proportion of 0.25% in patient samples versus 6.7% in control samples. This observation is suggestive of extensive antimicrobial susceptibility among the *Faecalibacterium*. A significant reduction in abundance of *Ruminococcus* spp. was observed in all patient groups compared to control microbiome abundances.

The metabolism of primary bile acids to secondary bile acids is classically performed by members of the order *Clostridiales*^19,23^. To determine if there was a link between secondary bile acid recovery and the microbiome, we measured the correlation between secondary bile acids and bacterial proportions for patient samples that were subjected to both analyses (*n* = 15). Total secondary bile acids correlated weakly (0.20 ≤ ρ < 0.4), with the observed proportion of *Clostridium sensus stricto* group 1 (ρ[15] = 0.205, *p* = 0.446). The genus *Clostridioides* was moderately correlated (0.40 ≤ ρ < 0.6) with DCA (ρ[15] = 0. 483, *p* = 0.068). Assessing the correlation between the abundances of bacterial genera and major secondary bile acid relative concentrations (DCA and total LCA/ILCA) (Fig. S4), we observed a strong correlation between DCA and *Agathobacter, Anaerovorax* and the *Eubacterium oxidoreducens* group across all patient samples with paired bile acid profiles and quantified microbiomes (Fig. S4). The genera *Anaerovorax, Gordonibacter, Romboutsia, Solobacterium* and, *Turicibacter* were strongly correlated with LCA and DCA. Four bacterial genera with moderate positive correlation (Fig. S4) were from the family *Lachnospiraceae.*

The genera within the *Lachnospiraceae* identified in this study were *Agathobacter*, *Anaerobutyricum* [*Eubacterium*] *hallii* group^31^, *Anaerocolumna*, *Anaerostignum*, *Anaerostipes*, *Blautia*, *Butyrivibrio*, *Coprococcus*, *Dorea*, *Eisenbergiella*, *Fusicatenibacter*, *Howardella*, *Hungatella*, *Lachnoanaerobaculum*, *Lachnoclostridium*, *Lachnospira*, *Mediterraneibacter* [*Ruminococcus*] *gnavus* group^32^, *Mediterraneibacter* [*Ruminococcus*] *torques* group, *Oribacterium*, *Pseudobutyrivibrio*, *Roseburia*, *Stomatobaculum* and *Tyzzerella*. There was no significant difference in the proportional representation of the *Lachnospiraceae* between patient groups (Fig. 4b). A shift in the composition of the *Lachnospiraceae* was observed when the constituent genera were examined (Fig. S4); Heterogeneous, *Agathobacter* dominant control profiles were replaced with *Lachnoclostridium* and *A*. *hallii* group bacteria in patient profiles (Fig. S5).

We examined the proportion of individual *Lachnospiraceae* genera for correlation with other bacteria groups in samples from day 8–10. Where a correlation was identified, *Ruminococcus* and *Solobacterium* were most often positively correlated to genera of *Lachnospiraceae.* There was a negative correlation between *Enterococcus* and *Anaerobutyricum* *hallii* group, *Dorea, Lachnoclostridium, Mediterraneibacter gnavus* group, and *Tyzzerella* (Τable S3)*.*

Comparing the median proportions of *Lachnospiraceae* across all major admission diagnoses we observed a decreased median proportion for respiratory infections, and non-respiratory infections. The difference was not significant after correction for multiple comparisons across six groups (Fig. S2c). Patients with respiratory infections had significantly lower proportions of *Lachnospiraceae* than healthy controls when compared to all other diagnoses (Fig. S2d). When patients were admitted for a non-respiratory infection, the *Lachnospiraceae* were also significantly decreased from the control group (Fig. S2e).

## Discussion

In this study, we identify divergent faecal bile acid profiles between patients and healthy controls; these differences may prove useful clinical markers of gut health and recovery. In our study group, day 1–3 and day 4–7 patient sampling time ranges had median primary bile acid compositions significantly above the control group while patients sampled during days 8–10 of PICU stay had bile acid compositions comparable to healthy control. The greater proportion of CA, and CDCA may be attributed to the reduction of intestinal bacteria capable of bile acid conversion^23,33–35^; concordantly, the inverse was observed for secondary bile acids. It is possible that the apparent recovery of bile acid metabolism by the end of PICU stays may reflects early-stage microbiome recovery. A future study with later timepoints would be needed to confirm this observation.

An alternative hypothesis for the increased levels of CA and CDCA in patients is an increase in the proportion of bacteria expressing bile salt hydrolase (BSH) in critically ill children.

This model would suggest that increased deconjugation of BAs leads to an over-abundance of primary BAs and a reduction in conjugated BAs in patients. BSHs are common among intestinal bacteria^18,36^, and an essential enzymatic process prior to the 7α/β-dehydratase pathway required to convert CA and CDCA to DCA and LCA/ILCA^19,37,38^. If an over-abundance of BSH expressing bacteria was increasing the concentration of primary BAs, one may expect to observe a decrease in conjugated BAs in faeces; no significant difference was observed in the proportion of conjugated BAs between timepoints. Our data suggests that the opposite may be true in some instances, where treatment for critical illness reduced the number of bacteria capable of de-conjugating bile salts. We observed individual patient samples containing high relative proportions of conjugated BAs where some samples contained proportions of TCA level reaching almost 80% and individual patient GCDCA reaching 50%. It was reported by Van Eldere and colleagues that deconjugation of TCA increased rates of primary to secondary bile acid conversion in mixed cultures^39^. Extrapolating this theory, one could assume an increased rate of deconjugation drives a conversion from primary to secondary bile acids if taurine conjugates were abundant. Taurine conjugated bile salts account for roughly 50% of the median conjugated primary bile salts in our analysis at each timepoint. BSHs are well represented in the genomes of gut bacteria^36^, and bacteria capable of a 7α/β-dehydratase reaction are rare^19,40^. If the bottleneck in primary to secondary bile acid metabolism is the rare 7α/β-dehydratase pathway, it would follow that a small relative increase in a BSH expressing bacterial population would not drive the difference in primary and secondary BAs a strongly as a decrease in bacteria capable of 7α/β-dehydration.

The composition of the gut microbiota was significantly affected during critical illness. Differences in microbial composition and richness between controls and critically ill patients were observed in this study. Decreasing alpha-diversities at varying body sites during hospitalisation in paediatric intensive care facilities has been previously reported by Rogers et al.^11^. An expected decrease in microbial richness, quantified by Shannon’s index, can be observed as the length of PICU stay increases. Treatment with broad-spectrum antimicrobials will be the driver for the compositional change and loss of diversity. Beta diversity varies widely in all treatment groups compared to control samples, and as expected there is a difference in faecal microbiome beta diversity between the control and sampling groups, most likely driven by antimicrobial treatment. A study by Chng and colleagues identified bacteria linked to microbiome recovery after antimicrobial therapy^12^. These RAB may act as keystone species^41,42^, providing metabolic substrates for endpoint fermenters such as the *Lachnospiraceae*. The general trend for RAB was to decrease in proportion with treatment length. The loss of these species in over time in our study is most likely explained by the duration of action of antimicrobial therapy. Decreased motility may account for some protection of RAB bacteria from antimicrobial killing, or the withdrawal of antibiotics but continued hospitalization may have allowed for recovery. Reduced gastric motility during critical illness can hinder sample collection. Patients in this study may not have had a first sample collected until 10 days post-admission.

Treatment for critical illness drives a compositional change in the *Lachnospiraceae*, with a slight decrease in median abundance. The observed increase in secondary bile acids in patients sampled at day 8–10 could be driven by a change in *Lachnospiraceae,* specifically, bacteria related to *Lachnoclostridium* and the *A. hallii* group bacteria^40,43,44^*.* Bacteria from *Lachnoclostridium* and the *A*. *hallii* group were present (> 10 counts) in 7 of 11 patient samples at days 8–10. Other bacteria in the family *Lachnospiraceae* were minor contributors in the microbiota of patients at days 8–10, with the exception of patient 18. Compositional changes of the *Lachnospiraceae* may be driven by changes in the upstream fermentation capacity of patient microbiota leading to increased production of mid-point fermentation products by the new dominant microbiota of critically ill children^10^. Bacteria in the *A. hallii* group and *Lachnoclostridium* are capable of utilising lactate produced by *Escherichia coli*, *Bifidobacterium*, *Lactobacillus*, and *Enterococcus*^45^*.* Further interpretation of the connection between bile acid production at days 8–10 and the microbiota is limited due to the small overlap in patients with both microbiomes and bile acid profiles (*n* = 2). An average PICU stay in the United Kingdom lasts 2–3 days, across all ages^46^. Samples collected at later timepoints are often from the sickest patients. The study is limited by the inability to follow the microbiome and bile acids of patients post-discharge.

Members of the *Lachnospiraceae* are known to metabolise primary to secondary bile acids through a 7α-dehydratase process^40^. The selection of enrichment for *A. hallii* group bacteria may be due to their ability to use both D- and L-lactate^45^. Increased total lactate was indicated as a measure of critical illness^10^. Members of the *Clostridiaceae* and *Ruminococcaceae* were not as abundant as the *Lachnospiraceae* at days 8–10, therefore unlikely to drive bile acid conversion.

An alternative hypothesis is that members of the *Lachnospiraceae* are differentially resistant to antibiotics, and strains are expanding to fill empty ecological niches. The interpretation of differential resistance is limited due to the use of 16S rRNA gene profiling and lack of information on the distribution of antimicrobial resistance genes. The shift in *Lachnospiraceae* composition from control to patient profiles appears as early as the first day of PICU admission. Duration of antimicrobial therapy and classes of antimicrobial can be difficult to quantify in a PICU setting; patients may have received antimicrobial therapy prior to admission, where information about treatment may held by another hospital and not readily available. After antimicrobial therapy certain bacteria recover more rapidly than others, contributing to the total biomass of faeces. We observed no strong effect due to change in faecal biomass, or biomass recovery when using contamination of 16S rRNA gene sequences derived from DNA extraction kits as a proxy for bacterial biomass.

During early life, the composition of the microbiome changes substantially, from species acquired during delivery, through breastfeeding and the transition to different foods. Studies have linked the method of delivery to an altered microbiome, children born by Caesarean section are at greater risk of autoimmune disease compared to children delivered via normal vaginal delivery^1,47–49^. As children mature, microbial complexity increases and “healthy” microbiomes are associated with greater bacterial diversity, with Bacteroidetes and Firmicutes making up the majority of the proportional representation^50^. The general trend in our study was that healthy children had richer microbial diversity and high proportions of Firmicutes and Bacteroidetes. Children requiring hospitalisation in PICUs often had highly disrupted microbial communities when compared to healthy children. Upon arrival to PICU, patients have most often received a course of antimicrobial therapy or will be treated with a broad-spectrum antimicrobial such as ceftriaxone. The effect of these treatments confounds the ability to interpret the impact of critical illness on the microbiome in the absence of internal reference.

Previously, we examined paediatric critical illness from a multi-component perspective and demonstrated that critical illness affects the microbiome of paediatric patients, which is consistent with other similar studies^10^. Faecal metabolic profiling indicated a higher correlation of sugars and lactate in patients, with short chain fatty acids increased in age matched controls. A brief examination of bile acids by Wijeyesekera et al*.*^10^ directly linked levels of the secondary bile acids DCA, LCA and ILCA to the proportion of *Bacteroides, Ruminococcus, Eubacterium, Lachnospiraceae*, and *Faecalibacterium* in faeces. Our results indicate a positive correlation in patient samples between secondary bile acids and the genera *Anaerovorax, Barnesiella, Faecalitalea, Gordonibacter, Megamonas, Odoribacter, Parasutterella, Pseudoflavonifractor, Solobacterium* and *Turicibacter,* however, the limited number of patient samples with both a quantified microbiome and a bile acid profile limits the predictive power of these observations.

Currently, profiling of microbiomes by 16S rRNA gene sequencing or shotgun metagenomics is a cost- and labour-intensive process. Even using only nanopore sequencing devices involves multiple steps. Days of data collection and processing are required to obtain a snapshot of the microbiome. The measurement of metabolomes by mass spectroscopy is rapid (under 30 min) and can be performed with minimal preparation from biological samples^10,51,52^. Clinical diagnostic laboratories may have services using mass spectroscopy as standard analytical techniques^53,54^. Measurement of primary and secondary BAs from blood, urine and faeces may therefore be within the capabilities of modestly outfitted laboratories.

The development of a healthy microbiome early in life is considered essential to systemic health^3,4,55,56^. If the microbiome fails to recover quickly, or enters an altered state, there may be differential bile acid metabolism, SCFA production, and overall altered microbial metabolic interactions. The recovery of the microbiota is dependent on the diversification of metabolic interactions after antibiotics^12,57^. Current predictive modelling programs attempt to predict host metabolomes from gut microbiomes^58,59^ and it may be possible to estimate the recovery of the microbiome based on metabolomics alone.

The power of this study to predict bacteria contributing to the recovery of bile acid profiles is limited by the overlap of patients with both microbiomes and bile acid profiles. The gastric transit of critically ill children is reduced due to multiple factors. The reduced intestinal motility limits the number and volume of faecal samples collected; therefore, analysis of some samples has been limited to bile acid profiling or 16S rRNA gene profiling.

## Conclusion

Our study observed relative concentrations of secondary faecal bile acids to be comparable with those of healthy controls after hospitalization in a PICU for 8–10 days. Compositional proportions of *Bacteroides*, *Ruminococcus*, and *Fecalibacterium* were reduced in microbiomes at day 8–10, but proportional representation of *Lachnospiraceae* remained close to control levels across all time ranges. Monitoring of faecal metabolites (SCFAs, sugars) and bile acids provides a rapid and cost-effective approach to determine intestinal microbiome health and recovery. Further studies are warranted to link markers of microbiome recovery after intervention for critical illness with clinical significance.

## Materials and methods

In this study we further examined the data collected by Wijeyesekera et al*.*^10^.

### Study participants

In this prospective, multi-centre cohort study, critically ill children were recruited if they were admitted to one of three paediatric intensive care units (PICUs) in the North Thames Region (UK). Following informed consent by caregivers, children aged between 1 and 16 years were eligible if they required mechanical ventilation. Children with chronic illness or prolonged steroid use were excluded. Healthcare data was acquired from hospital medical records. Patients receiving enteral nutrition were given expressed breast milk or formula without probiotic supplementation. Standard antimicrobial treatment upon admission to PICU is the administration of a broad-spectrum agent, often a third-generation cephalosporin unless otherwise clinically indicated. In order to compare data with healthy controls, faecal samples were obtained from age-matched children, who were recruited from the local community. These children were eligible if they were free of intercurrent illness, had a normal healthy diet, and had not received antibiotics in the prior 3 months.

Approval for the study was granted by the East Midlands-Nottingham 2 Research Ethics Committee for recruitment from the PICUs at Cambridge University Hospitals NHS Foundation Trust, Great Ormond Street Hospital NHS Foundation Trust, and Imperial College Healthcare NHS Foundation Trust. The City Road and Hampstead Research Ethics committee approved recruitment of healthy children in the Cambridge vicinity. REC reference: 13/LO/0974. All methods were performed in accordance with the relevant guidelines and regulations.

### Faecal samples

Faecal samples were collected at early (within first 3 days of PICU admission), mid (days 4–7 of PICU admission), and late timepoints (days 8–10 of PICU admission) from critically ill children. Serial samples were collected from each child as they became available. Samples were used for 16S rRNA gene sequencing, and or bile acid profiling as biomass allowed. A single faecal sample was collected from healthy children. All samples from critically ill children were taken from nappies, placed in sterile plastic containers, and stored at − 70 °C until use.

### Taxonomic data

We performed sequencing of the bacterial 16S rRNA gene in the faecal samples and allocated these data into the various taxonomic groups, as described previously by Wijeyesekera et al*.*^10^. Briefly, total nucleic acid was extracted from faecal samples using the PowerFecal DNA Isolation Kit (MPBio). The V1V2 region of the bacterial 16S rRNA gene was amplified by PCR and sequenced using a MiSeq V3 600 cycle flow cell (Illumina) at the Wellcome Sanger Institute, UK. 16S rRNA gene sequences were quality filtered and analysed using Mothur software^60^.

### Bile acid analysis

Bile acids were extracted from faeces and assayed using liquid chromatography-mass spectrometry (LC–MS), following the methods described by Sarafian et al*.*^51^. Briefly, faecal samples were processed to extract faecal water, and the supernatant was mixed 1:3 with ice-cold methanol as described previously by Gratton et al.^10,61^ . Reverse-phase chromatography was performed using two mobile phases and a short alkyl (C8) stationary phase adapted from Shockcor et al*.*^62^. Mobile phase A consisted of acetonitrile and ultra-pure water (1:10), with a final concentration of 1 mM ammonium acetate and adjusted to pH 4.18 using acetic acid, and mobile phase B consisted of acetonitrile and 2-propanol (1:1). Bile acid identification was based on the retention times of individual standards. LC–MS settings are published in Sarafian et al*.*^51^. Example LC–MS traces are available in Fig. S6 and individual bile acid retention times are summarised in Table S4.

### Statistical analysis

Data analysis was performed using R v4.0.2^63^. Non-parametric variance was calculated using the Kruskal–Wallis rank sum test^64^. Conover-Iman rank sum tests were used to perform multiple comparisons when H_0_ is rejected by a Kruskal–Wallis test^65^. Inter-group significance displayed in graphs was calculated using the Conover-Iman test. Conover-Iman tests were performed using the conover.test package for R^66^. Multiple comparisons were corrected using the method proposed by Benjamini and Hochberg, as implemented in conover.test^66,67^. Graphs were created using ggplot2^68^. Data frame melting was performed with reshape2^69^. Weight-for-Age Z-scores were calculated using zscorer^70^. Values which were not calculated by the R package were calculated with PediTools^71^. Shannon diversity indices were calculated using the diversity function from vegan 2.5–6^72^. Beta-diversity was calculated using the vegdist function of vegan and plotted with ggplot2. Spearman’s ρ was calculated using cor.test in R^63^. Correlation interpretations are based on guidelines published by the British Medical Journal^73^.

### Ethics approval and consent to participate

Approval for the study was granted by the East Midlands-Nottingham 2 Research Ethics Committee for recruitment from the PICUs at Cambridge University Hospitals NHS Foundation Trust, Great Ormond Street Hospital NHS Foundation Trust, and Imperial College Healthcare NHS Foundation Trust. The City Road and Hampstead Research Ethics committee approved recruitment of healthy children in the Cambridge vicinity. REC reference: 13/LO/0974.

### Consent for Publication

Parents of all study subjects provided consent for publication of de-identified data.

## Supplementary Information

Supplementary Information 1.

Supplementary Information 2.

Supplementary Information 3.

Supplementary Information 4.

Supplementary Information 5.

Supplementary Information 6.

Supplementary Information 7.

Supplementary Information 8.

Supplementary Information 9.

Supplementary Information 10.

Supplementary Information 11.

## Data Availability

The datasets analysed during the current study are available in the European Nucleotide Archive, https://www.ebi.ac.uk/ena/browser/view/prjeb13830. Data used for analysis can be found in the Supplementary Information 1: Data table, and Supplementary Information 2: Family Taxonomy.

## Abbreviations

12-DHCA: 12-Dehydrocholic acid
23 nor-5β-ClA,3α,12α-diol: 23-Nor,5ß-Cholanic acid 3α-,12α-diol
3-DHCA: 3-Dehydrocholica acid
3-KClA: 3-Ketocholanic acid
312-diKClA: 3,12-Diketocholanic acid
3α-H12-KLCA: 3α-Hydroxy,12-Ketolithocholic acid
3α-H12-KLCA: 3α-Hydroxy,12-Ketolithocholic acid
3α-H6: 7DiKClA, 3α-Hydroxy,6-,7-Diketo-Cholanic acid
5β-ClA,3β12A-diol: 5ß-Cholanic acid 3ß-,12α-diol
CDCA: Chenodeoxycholic acid
CA: Cholic acid
DCA: Deoxycholic acid
GCDA: Glycochenodeoxycholic acid
GUDCA: Glycoursodeoxycholic acid
IQR: Inter-quartile range
ILCA: Isolithocholic acid
LC–MS: Liquid Chromatography-Mass Spectrometery
LCA: Lithocholic acid
PICUs: Paediatric intensive care units
RAB: Recovery Associated Bacteria
RC: Relative concentrations
SCFA: Short Chain Fatty Acid
TCA: Taurocholic acid
THCA: Taurohyocholic acid
HSD: Tukey’s Honest Significant Differences
UDCA: Ursodeoxycholic acid

## References

[1] Shao Y (2019). Stunted microbiota and opportunistic pathogen colonization in caesarean-section birth. Nature.

[2] Milani C (2017). The first microbial colonizers of the human gut: composition, activities, and health implications of the infant gut microbiota. Microbiol. Mol. Biol. Rev..

[3] Langdon A, Crook N, Dantas G (2016). The effects of antibiotics on the microbiome throughout development and alternative approaches for therapeutic modulation. Genome Med..

[4] Arrieta MC, Stiemsma LT, Amenyogbe N, Brown E, Finlay B (2014). The intestinal microbiome in early life: Health and disease. Front. Immunol..

[5] Stewart CJ (2018). Temporal development of the gut microbiome in early childhood from the TEDDY study. Nature.

[6] Ruohtula T (2019). Maturation of gut microbiota and circulating regulatory T cells and development of IgE sensitization in early life. Front. Immunol..

[7] Heida FH (2016). A necrotizing enterocolitis-associated gut microbiota is present in the meconium: results of a prospective study. Clin. Infect. Dis..

[8] Rinninella E (2019). What is the healthy gut microbiota composition? A changing ecosystem across age, environment, diet, and diseases. Microorganisms.

[9] Dickson RP (2016). The microbiome and critical illness. Lancet Respir. Med..

[10] Wijeyesekera A (2019). Multi-compartment profiling of bacterial and host metabolites identifies intestinal dysbiosis and its functional consequences in the critically Ill child. Crit. Care Med..

[11] Rogers MB (2016). Disruption of the microbiota across multiple body sites in critically ill children. Microbiome.

[12] Chng KR (2020). Metagenome-wide association analysis identifies microbial determinants of post-antibiotic ecological recovery in the gut. Nat. Ecol. Evol..

[13] Moron R (2019). The importance of the microbiome in critically ill patients: Role of nutrition. Nutrients.

[14] Moreno-Indias I (2015). Intermittent hypoxia alters gut microbiota diversity in a mouse model of sleep apnoea. Eur. Respir. J..

[15] Looft T (2012). In-feed antibiotic effects on the swine intestinal microbiome. Proc. Natl. Acad. Sci..

[16] Schubert K, Olde Damink SWM, von Bergen M, Schaap FG (2017). Interactions between bile salts, gut microbiota, and hepatic innate immunity. Immunol. Rev..

[17] Urdaneta V, Casadesús J (2017). Interactions between bacteria and bile salts in the gastrointestinal and hepatobiliary tracts. Front. Med..

[18] Heinken A (2019). Systematic assessment of secondary bile acid metabolism in gut microbes reveals distinct metabolic capabilities in inflammatory bowel disease. Microbiome.

[19] Ridlon JM, Kang DJ, Hylemon PB (2006). Bile salt biotransformations by human intestinal bacteria. J. Lipid Res..

[20] Kang JD (2019). Bile acid 7α-dehydroxylating gut bacteria secrete antibiotics that inhibit clostridium difficile: role of secondary bile acids. Cell Chem. Biol..

[21] Solbach P (2018). BaiCD gene cluster abundance is negatively correlated with Clostridium difficile infection. PLoS ONE.

[22] Staley C, Weingarden AR, Khoruts A, Sadowsky MJ (2017). Interaction of gut microbiota with bile acid metabolism and its influence on disease states. Appl. Microbiol. Biotechnol..

[23] Ridlon JM, Kang DJ, Hylemon PB, Bajaj JS (2014). Bile acids and the gut microbiome. Curr. Opin. Gastroenterol..

[24] Ridlon JM, Wolf PG, Gaskins HR (2016). Taurocholic acid metabolism by gut microbes and colon cancer. Gut Microbes.

[25] Ma H, Patti ME, Endocrinologist A (2014). Bile acids, obesity, and the metabolic syndrome. Best Pract. Res. Clin. Gastroenterol..

[26] Festi D (2014). Gut microbiota and metabolic syndrome. World J. Gastroenterol..

[27] Reddy BS, Narasawa T, Weisburger JH, Wynder EL (1976). Promoting effect of sodium deoxycholate on colon adenocarcinomas in germfree Rats2. JNCI J. Natl. Cancer Inst..

[28] Reddy BS, Watanabe K, Weisburger JH, Wynder EL (1977). Promoting effect of bile acids in colon carcinogenesis in germ-free and conventional F344 rats. Cancer Res..

[29] Attene-Ramos MS, Wagner ED, Gaskins HR, Plewa MJ (2007). Hydrogen sulfide induces direct radical-associated DNA damage. Mol. Cancer Res..

[30] Devkota S (2012). Dietary-fat-induced taurocholic acid promotes pathobiont expansion and colitis in Il10-/- mice. Nature.

[31] Shetty SA (2018). Reclassification of Eubacterium hallii as Anaerobutyricum hallii gen. nov., comb. Nov., and description of Anaerobutyricum soehngenii sp. Nov., a butyrate and propionate-producing bacterium from infant faeces. Int. J. Syst. Evol. Microbiol..

[32] Togo, A. H. *et al.* Description of Mediterraneibacter massiliensis, gen. nov., sp. nov., a new genus isolated from the gut microbiota of an obese patient and reclassification of Ruminococcus faecis, Ruminococcus lactaris, Ruminococcus torques, Ruminococcus gnavus and Clostridium glycyrrhizinilyticum as Mediterraneibacter faecis comb. nov., Mediterraneibacter lactaris comb. nov., Mediterraneibacter torques comb. nov., Mediterraneibacter gnavus comb. nov. and Mediterraneibacter glycyrrhizinilyticus comb. nov. *Antonie Van Leeuwenhoek* **111**, 2107–2128 (2018). 10.1007/s10482-018-1104-y 29855844

[33] Hirano S, Nakama R, Tamaki M, Masuda N, Oda H (1981). Isolation and characterization of thirteen intestinal microorganisms capable of 7α-dehydroxylating bile acids. Appl. Environ. Microbiol..

[34] Berr F, Kullak-Ublick GA, Paumgartner G, Monzing W, Hylemon PB (1996). 7α-Dehydroxylating bacteria enhance deoxycholic acid input and cholesterol saturation of bile in patients with gallstones. Gastroenterology.

[35] Chiang JYL (2009). Bile acids: regulation of synthesis. J. Lipid Res..

[36] Song Z (2019). Taxonomic profiling and populational patterns of bacterial bile salt hydrolase (BSH) genes based on worldwide human gut microbiome. Microbiome.

[37] Stellwag EJ, Hylemon PB (1979). 7alpha-Dehydroxylation of cholic acid and chenodeoxycholic acid by Clostridium leptum. J. Lipid Res..

[38] Batta AK (1990). Side chain conjugation prevents bacterial 7-dehydroxylation of bile acids. J. Biol. Chem..

[39] Van Eldere J, Celis P, De Pauw G, Lesaffre E, Eyssen H (1996). Tauroconjugation of cholic acid stimulates 7 alpha-dehydroxylation by fecal bacteria. Appl. Environ. Microbiol..

[40] Vital M, Rud T, Rath S, Pieper DH, Schlüter D (2019). Diversity of bacteria exhibiting bile acid-inducible 7α-dehydroxylation genes in the human gut. Comput. Struct. Biotechnol. J..

[41] Gibbons SM (2020). Keystone taxa indispensable for microbiome recovery. Nat. Microbiol..

[42] Paine RT (1995). A conversation on refining the concept of keystone species. Conserv. Biol..

[43] Begley M, Hill C, Gahan CGM (2006). Bile salt hydrolase activity in probiotics. Appl. Environ. Microbiol..

[44] Marion S (2018). In vitro and in vivo characterization of Clostridium scindens bile acid transformations. Gut Microbes.

[45] Duncan SH, Louis P, Flint HJ (2004). Lactate-utilizing bacteria, isolated from human feces, that produce butyrate as a major fermentation product. Appl. Environ. Microbiol..

[46] PICANet_2019_Annual_Report_Tables_and_Figures_v1.0.pdf.

[47] Neu J, Rushing J (2011). Cesarean versus vaginal delivery: long-term infant outcomes and the hygiene hypothesis. Clin. Perinatol..

[48] Biasucci G, Benenati B, Morelli L, Bessi E, Boehm G (2008). Cesarean delivery may affect the early biodiversity of intestinal bacteria. J. Nutr..

[49] Dominguez-Bello MG (2016). Partial restoration of the microbiota of cesarean-born infants via vaginal microbial transfer. Nat. Med..

[50] Yassour M (2016). Natural history of the infant gut microbiome and impact of antibiotic treatment on bacterial strain diversity and stability. Sci. Transl. Med..

[51] Sarafian MH (2015). Bile acid profiling and quantification in biofluids using ultra-performance liquid chromatography tandem mass spectrometry. Anal. Chem..

[52] Kakiyama G (2014). A simple and accurate HPLC method for fecal bile acid profile in healthy and cirrhotic subjects: Validation by GC-MS and LC-MS. J. Lipid Res..

[53] Strathmann FG, Hoofnagle AN (2011). Current and future applications of mass spectrometry to the clinical laboratory. Am. J. Clin. Pathol..

[54] Seger C, Salzmann L (2020). After another decade: LC–MS/MS became routine in clinical diagnostics. Clin. Biochem..

[55] Bokulich NA (2016). Antibiotics, birth mode, and diet shape microbiome maturation during early life. Sci. Transl. Med..

[56] Wang C (2007). Effects of oral administration of Bifidobacterium breve on fecal lactic acid and short-chain fatty acids in low birth weight infants. J. Pediatr. Gastroenterol. Nutr..

[57] Suez J (2018). Post-antibiotic gut mucosal microbiome reconstitution is impaired by probiotics and improved by autologous FMT. Cell.

[58] Mallick H (2019). Predictive metabolomic profiling of microbial communities using amplicon or metagenomic sequences. Nat. Commun..

[59] Larsen PE, Dai Y (2015). Metabolome of human gut microbiome is predictive of host dysbiosis. GigaScience.

[60] Schloss PD (2009). Introducing mothur: Open-source, platform-independent, community-supported software for describing and comparing microbial communities. Appl. Environ. Microbiol..

[61] Gratton J (2016). Optimized Sample Handling Strategy for Metabolic Profiling of Human Feces. Anal. Chem..

[62] Shockcor, J., Crowe, H., Yu, K. & Shion, H. Analysis of Intact Lipids from Biologics Matrices by UPLC / Ion Mobility TOF-MS. *Waters Corp. Milford MA USA* 2–4 (2011).

[63] R Core Team. R software: Version 4.0.2. *R Found. Stat. Comput.* (2020).

[64] Kruskal WH, Wallis WA (1952). Use of ranks in one-criterion variance analysis. J. Am. Stat. Assoc..

[65] Conover WJ, Iman RL (1981). Rank transformations as a bridge between parametric and nonparametric statistics. Am. Stat..

[66] Dinno, A. conover.test: Conover-Iman Test of Multiple Comparisons Using Rank Sums. (2017).

[67] Benjamini Y, Hochberg Y (1995). Controlling the false discovery rate: a practical and powerful approach to multiple testing. J. R. Stat. Soc. Ser. B Methodol..

[68] Wickham H (2016). ggplot2 Elegant Graphics for Data Analysis (Use R!). Springer.

[69] Wickham, H. R: Package ‘reshape2’. *CRAN* (2017).

[70] Guevarra, E. *nutriverse/zscorer: zscorer v0.3.1*. (Zenodo, 2019). 10.5281/zenodo.3510075.

[71] Chou JH, Roumiantsev S, Singh R (2020). PediTools electronic growth chart calculators: applications in clinical care, research, and quality improvement. J. Med. Internet Res..

[72] Oksanen, J. *et al.* vegan: Community Ecology Package. R package version 2.5–2. *Cran R* (2019).

[73] 11. Correlation and regression | The BMJ. *The BMJ | The BMJ: leading general medical journal. Research. Education. Comment* https://www.bmj.com/about-bmj/resources-readers/publications/statistics-square-one/11-correlation-and-regression (2020).

