# Supplementary figures and images for: Profiling gut microbiota and bile acid metabolism in critically ill children
Source: Sci Rep. 2022 Jun 21;12:10432. doi: 10.1038/s41598-022-13640-0 (PMC9213539; doi:10.1038/s41598-022-13640-0)

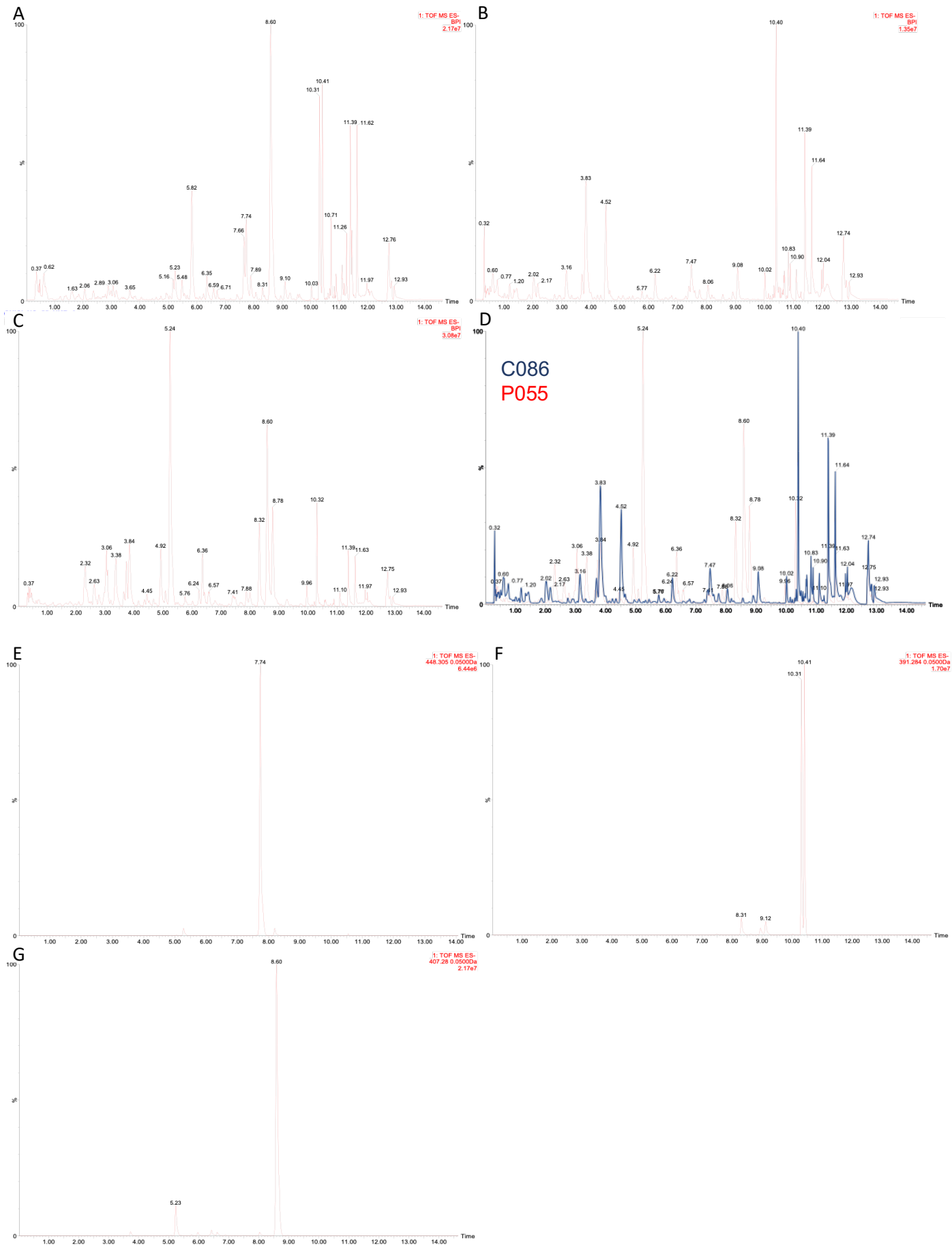

Supplement: Supplementary file 8 — Supplementary Information 8. [file 41598_2022_13640_MOESM8_ESM.pdf]
