# Supplementary material for: Profiling gut microbiota and bile acid metabolism in critically ill children
Source: Sci Rep. 2022 Jun 21;12:10432. doi: 10.1038/s41598-022-13640-0 (PMC9213539; doi:10.1038/s41598-022-13640-0)
Supplement: Supplementary file 10 — Supplementary Information 10. [file 41598_2022_13640_MOESM10_ESM.docx]

Table S1: Primary organ failure upon admission to PICU and survival of patients by admission

| **Primary Organ Failure** | **Number (%)** | **Survival (%)** |
| --- | --- | --- |
| Neurological | 14 (20.6) | 14/14 (100) |
| Respiratory | 21 (30.9) | 19/21 (90.5) |
| Sepsis | 27 (39.7) | 27/27 (100) |
| Trauma/Surgical | 6 (8.8) | 5/6 (83.3) |
| TOTAL | 68 (100) | 65/68 (95.5) |

Table S2: Antimicrobial usage for patients with microbiome profiles. Antibiotics are grouped by structural class. Inhibitors are Tazobactam and Clavulanic acid.

| **Antimicrobial/Inhibitor** | **Patients (%)** |
| --- | --- |
| Aminoglycosides | 10 (23.3) |
| Amphenicols | 1 (2.3) |
| Cephalosporins | 28 (65.1) |
| Glycopeptides | 3 (7.0) |
| Lincosamides | 8 (9.3) |
| Macrolides | 11 (25.6) |
| Metronidazole | 2 (4.7) |
| Penicillins | 15 (34.9) |
| Quinolones | 2 (4.7) |
| β-lactam inhibitors | 13 (30.2) |

Table S3:Spearman’s rank correlation (rho) betwee Lachnospiraceae bacteria counts and other bacterial counts in samples collected on days 8-10 of hospitalization. Eleven samples were collected from hospitalized children during days 8-10. Bacteria with negative correlations to are underlined. Values of p ≤ 0.05 are in **Bold.**

| **Genera** | **Correlated Genera** | **⍴[11]** | **p-value** |
| --- | --- | --- | --- |
| *Agathobacter* | *Anaerobutyricum* *hallii* group | 0.528 | 0.0950 |
| *Anaerobutyricum* *hallii* group | *Ruminococcus* | 0.544 | 0.0834 |
| *Anaerobutyricum* *hallii* group | *Lachnoclostridium* | 0.539 | 0.0874 |
| *Anaerobutyricum* *hallii* group | *Enterococcus* | **-0.768** | **0.0058** |
| *Anaerobutyricum* *hallii* group | *Faecalibacterium* | **0.930** | **3.43e-05** |
| *Coprococcus* | *Anaerobutyricum* *hallii* group | **0.634** | **0.0361** |
| *Dorea* | *Enterococcus* | -0.543 | 0.0840 |
| *Eisenbergiella* | *Ruminococcus* | 0.553 | 0.0776 |
| *Eisenbergiella* | *Lachnoclostridium* | **0.659** | **0.0273** |
| *Fusicatenibacter* | *Anaerobutyricum* *hallii* group | **0.830** | **0.0016** |
| *Hungatella* | *Ruminococcus* | **0.819** | **0.0021** |
| *Hungatella* | *Anaerobutyricum* *hallii* group | 0.533 | 0.0911 |
| *Hungatella* | *Lachnoclostridium* | **0.961** | **2.650E-06** |
| *Hungatella* | *Solobacterium* | 0.562 | 0.0720 |
| *Lachnoclostridium* | *Ruminococcus* | **0.861** | **6.631E-04** |
| *Lachnoclostridium* | *Escherichia-Shigella* | 0.560 | 0.0730 |
| *Lachnoclostridium* | *Enterococcus* | -0.528 | 0.0947 |
| *Lachnoclostridium* | *Solobacterium* | 0.539 | 0.0872 |
| *Mediterraneibacter* *gnavus* group | *Ruminococcus* | **0.649** | **0.0306** |
| *Mediterraneibacter* *gnavus* group | *Anaerobutyricum* *hallii* group | **0.687** | **0.0194** |
| *Mediterraneibacter* *gnavus* group | *Enterococcus* | -0.560 | 0.0731 |
| *Mediterraneibacter* *torques* group | *Ruminococcus* | **0.758** | **0.0068** |
| *Mediterraneibacter* *torques* group | *Anaerobutyricum* *hallii* group | **0.731** | **0.0106** |
| *Mediterraneibacter* *torques* group | *Lachnoclostridium* | 0.527 | 0.0958 |
| *Oribacterium* | *Bifidobacterium* | -0.547 | 0.0814 |
| *Pseudobutyrivibrio* | *Ruminococcus* | 0.560 | 0.0727 |
| *Pseudobutyrivibrio* | *Bifidobacterium* | 0.533 | 0.0913 |
| *Pseudobutyrivibrio* | *Streptococcus* | **0.688** | **0.0192** |
| *Roseburia* | *Ruminococcus* | **0.654** | **0.0289** |
| *Roseburia* | *Anaerobutyricum hallii* group | **0.664** | **0.0257** |
| *Tyzzerella* | *Bacteroides* | 0.558 | 0.0744 |
| *Tyzzerella* | *Ruminococcus* | **0.642** | **0.0331** |
| *Tyzzerella* | *Escherichia-Shigella* | 0.579 | 0.0619 |
| *Tyzzerella* | *Anaerobutyricum* *hallii* group | **0.679** | **0.0216** |
| *Tyzzerella* | *Lachnoclostridium* | **0.760** | **0.0067** |
| *Tyzzerella* | *Enterococcus* | **-0.779** | **0.0047** |
| *Tyzzerella* | *Solobacterium* | **0.707** | **0.0150** |

*Table S4: Mass/Charge ratios and retention times for bile acid standards.*

| **Molecule** | **Abbreviation** | **m/z** | **Retention Time** |
| --- | --- | --- | --- |
| Taurohyocholic Acid | THCA | 514.2847 | 4.29 |
| 3α-Hydroxy-6,7-DiketoCholanic Acid | 3α-H,6,7diKClA | 403.2477 | 4.49 |
| Taurocholic Acid | TCA | 514.2852 | 5.15 |
| Glycoursodeoxycholic Acid | GUDCA | 448.3051 | 5.26 |
| 12-Dehydrocholic Acid | 12-DHCA | 405.2637 | 6.34 |
| 3-Dehydrocholic Acid | 3-DHCA | 405.2638 | 7.43 |
| Glycochenodeoxycholic Acid | GCDCA | 448.3062 | 7.71 |
| 3,12-Diketocholanic Acid | 3,12-diKClA | 387.2531 | 8.05 |
| Ursodeoxycholic Αcid | UDCA | 391.2840 | 8.3 |
| Cholic acid | CA | 407.2795 | 8.57 |
| 3α-Hydroxy-12 Ketolithocholic Acid | 3α-H,12-KLCA | 389.2688 | 9.09 |
| 5ß-Cholanic Acid-3ß, 12α-diol | 5β-ClA,3β12α-diol | 391.2219 | 9.1 |
| 23-nor-5β-Cholanic Acid-3α, 12α-diol | 23 nor-5β-ClA,3α,12α-diol | 377.2681 | 9.73 |
| Chenodeoxycholic Acid | CDCA | 391.2843 | 10.3 |
| Deoxycholic Acid | DCA | 391.2844 | 10.4 |
| Isolithocholic Acid | ILA | 375.2893 | 10.5 |
| 3-Ketocholanic Acid | 3-KClA | 373.2738 | 10.7 |
| Lithocholic acid | LCA | 375.2893 | 10.83 |
