# Supplementary material for: Profiling gut microbiota and bile acid metabolism in critically ill children
Source: Sci Rep. 2022 Jun 21;12:10432. doi: 10.1038/s41598-022-13640-0 (PMC9213539; doi:10.1038/s41598-022-13640-0)
Supplement: Supplementary file 11 — Supplementary Information 11. [file 41598_2022_13640_MOESM11_ESM.docx]

**Supplementary Figure Legends**

*Figure S1: Individual bile acids as relative concentrations (RC) of total bile acid detected in faecal water. A)* 3α*-*Hydroxy,6-,7-Diketo-Cholanic acid (3a-H,6,7diKClA), b) Taurohyocholic acid (THCA), c) Glycoursodeoxycholic acid (GUDCA), d) 3-Dehydrocholica acid (3-DHCA) e) 3,12-Diketocholanic acid (3,12-diKClA) f) Ursodeoxycholic acid(UDCA), g) 12-Dehydrocholic acid (12-DHCA) h) Glycochendeoxycholic acid (GCDA) i) 3α-Hydroxy,12-Ketolithocholic acid (3α-H,12-KLCA), j) Isolithocholic acid(ILCA), k) Chendeoxycholic acid (CDCA), l) 5ß-Cholanic acid 3ß-,12α-diol (5b-ClA,3b12A-diol) m) 3-Ketocholanic acid (3-KClA) n) Lithocholic acid (LCA), o) Deoxycholic_acid (DCA), p) Cholic acid (CA), q) 23-nor,5ß-Cholanic Acid 3α-,12α-diol (23 nor-5b-ClA,3a,12a-diol) r) 3α-Hydroxy,12-Ketolithocholic acid (3α-H,12-KLCA), s) Taurocholic acid (TCA). Conover-Iman significance *** p ≤0.001, ** p ≤0.01, * p ≤0.05, p>0.05 = not shown.

*Figure S2: Supplemental analysis*. a) RAB proportion versus serial sample number, Point shapes= Triangles =sample taken on PICU, Square = Sample taken on General ward. Point colour = sampling timepoint. Red = day 1-3, Orange = day 4-7, Green = days 8-10. A significant reduction in the proportion of RAB was observed in the first patient sample collected (30% RAB, n=43, day 1-3=20, day 4-7=15, days 8-10=8) relative to control samples (60% RAB, n=43) (p=0.002). The median RAB proportion observed in second samples was 0.27% (n=7, day 1-3=1, day 4-7=4, days 8-10=2), which was also significantly reduced from median control proportion of RAB. A difference between first and second samples was observed but was not significant. Only one patient had 3 serial samples analysed, with the third sample containing 0.2% RAB. B) RAB proportion versus serial primary diagnosis. C) Lachnospiraceae proportion versus primary diagnosis, D) Lachnospiraceae proportion versus respiratory disease, E) Lachnospiraceae versus admission for infection versus non-infection. Conover-Iman significance *** p ≤0.001, ** p ≤0.01, * p ≤0.05, p>0.05 = not shown.

*Figure S3: Recovery associated bacteria (RAB) identified by 16s rRNA gene sequencing.* Proportions of previously describe RAB which were identified in this study. a) Proportion of *Alistipes*, b) Proportion of *Bacteroide,* c) Proportion of Bifidobacterium, d) Proportion of Coprococcus e) Proportion of *Desulfovibrio*, f) Proportion of *Faecalibacterium,* g) Proportion of *Parabacteroides* h) Proportion of *Roseburia,* i) Proportion of *Ruminococcus,* j) Proportion of *Subdoligranulum.* Conover-Iman significance *** p ≤0.001, ** p ≤0.01, * p ≤0.05, p>0.05 = not shown.

*Figure S4: Heatmap of Spearman’s Ρ* *for correlation between bile acid proportion and bacterial relative abundance.* Spearman’s Rho is represented on a scale from blue (-1.0) to red (1.0) statistical significance is displayed for each comparison. Comparisons are based on 15 samples from patients who had both a bile acid profile and a 16S rRNA gene profile. Significance *** p ≤0.001, ** p ≤0.01, * p ≤0.05, p>0.05 = not shown.

*Figure S5: Composition of Lachnospiraceae in faecal samples.* Raw counts for all *Lachnospiraceae* identified in this study. Lachnospiraceae identified in the control groups are highly mixed populations. Predominant control genera are the *Agathobacter, Coprococcus, Mediterranibacter torques* group, and *Roseburia.* Patient microbiomes are different from day 1 of hospitalization. Genera of the *Lachnospiraceae* which are highly represented in patient samples are the *Anaerobutrycum hallii* group, *Lachnoclostridium,* and *Mediterranibacter torques* group.

*Figure S6: Example LC-MS traces of bile acids. Mass spectroscopic detection of bile acids versus liquid chromatographic retention time. A) Pooled control sample inclusive of bile acids and salts from Table S6. B) Trace from Control subject C086. C) Trace from critically ill subject P055. D) Overlay of C086 (blue), and P055 (red). Major compositional bile acid peaks differ between healthy controls and patients. E) Trace for Glycochenodeoxycholic acid (GCDCA). F) Trace for Chenodeoxycholic acid (CDCA), Deoxycholic acid (DCA), and Ursodeoxycholic acid (UDCA). G) Trace for Cholic acid (CA).*

**Additional files**

1. **Kean_2021_R_scripts.Rmd** – R Markdown file containing the code used to produce the above analysis. Script is based on R 4.0.2. Analysis requires *Kean_2021_data_table.csv* and *Kean_2021_family_taxonomy.csv*
2. **Kean_2021_data_table.csv** – Data Table – Comma separated variable file containing anonymised data on which the above analysis was based.
3. **Kean_2021_family_taxonomy.csv** – Comma separated variable file containing 16s rRNA gene counts separated taxonomically at the Family level based on the SILVA138.1 SSU database. The 10 most abundant families from each of the control and patient microbiomes are included.
4. **Kean_2021_Supplementary_tables.pdf** – Supplementary tables 1-4 Extended patient demographics, Correlation of *Lachnospiracieae* to identified bacterial genera, and Mass/Charge ratios and retention times for bile acid standards.
5. **Kean_2021_Figure_S1.eps** – Figure S1, Individual bile acids as relative concentrations (RC) of total bile acid detected in faecal water
6. **Kean_2021_Figure_S2.eps** – Figure S2, Supplemental analysis of data
7. **Kean_2021_Figure_S3.eps** - Figure S3, Recovery associated bacteria (RAB) identified by 16s rRNA gene sequencing
8. **Kean_2021_Figure_S4.eps –** Figure S4, Heatmap of Spearman’s Ρ for correlation between bile acid proportion and bacterial relative abundance
9. **Kean_2021_Figure_S5.eps** – Figure S5, Composition of *Lachnospiraceae* in faecal samples
10. **Kean_2021_Figure_S6.pdf**  - Figure S6, Example LC-MS traces of bile acids.
